# Science is innate!

**DOI:** 10.1186/s13059-014-0477-0

**Published:** 2014-10-02

**Authors:** Jack A Gilbert

**Affiliations:** Institute for Genomic and Systems Biology, Argonne National Laboratory, 9700 South Cass Avenue, Argonne, IL 60439 USA; Department of Ecology and Evolution, University of Chicago, 1101 E 57th Street, Chicago, IL 60637 USA; Marine Biological Laboratory, 7 MBL Street, Woods Hole, MA 02543 USA; College of Environmental and Resource Sciences, Zhejiang University, Hangzhou, 310058 China

I am a professional scientist. What does that mean? I think it is safe to say it means that, to the best of my ability, I apply the scientist method to test hypotheses and gain a clearer understanding of the world around me. However, I am also a human being, and so when I am asked, as I often am, about how my research findings have influenced my day-to-day activities, I like to take a step back and think about what it means to be a scientist.

Science is a method for questioning, observing and interpreting; it helps to focus enquiry, refine models and generate evidence to test hypotheses. We generate a hypothesis, for example, ‘I have enough gas (petrol) to get to work this morning’, as a statement of ‘fact’ that needs to be tested. To define the potential outcomes of the hypothesis a model is generated, which helps us to predict if the hypothesis is true or false. In this case, our measured variables would be the gas gauge, the known distance to work, the time of day and whether there will be traffic, the potential for road works that could cause diversions, and whether you need to stop on the way to work for coffee or to drop off dry cleaning. By taking into consideration all these variables you are able to make an informed prediction as to whether your hypothesis will be validated or falsified. I can also account for highly improbable events by rationalizing that ‘even if something does happen, I am aware of the locations of gas stations on each major route to and from work’ (or my GPS will tell me). Obviously, the proof is in the pudding; you have a hypothesis and a model that has generated a prediction based on probabilities, and to determine whether your prediction is right today, you have only got to go out there and do the actual experiment and observe its outcome.

These ‘mental gymnastics’ are performed without really considering what we are doing. The problem-solving ability that we take for granted is the foundation of the scientific method, an innate force that helps us to build on knowledge and understanding. The same principle works for cooking a meal, getting the kids to school, going on vacation, attending talks at a conference, or any of the numerous normal things that we all do every day.

In my work exploring the microbial ecology of humans and their environments, it has become self-evident that bacteria influence many aspects of our lives, and that disturbing the relationship between our body and our bacteria can have serious consequences. While the majority of the causal evidence for this relationship has been performed in animal studies, I think it is reasonably safe to assume that these relationships probably apply to us as well. Either way, elegant research showing that disturbing the microbiota of mice can affect the propensity towards obesity [1] and allergies [2] is bound to influence not just our health, but how we approach our life styles in many ways.

Our own unpublished work on the mechanisms by which changes in gut microbial communities alter adipose tissue generation in mice, has definitely altered my life. Longitudinal sampling of the mouse microbiome over diurnal timescales suggested that the daily timings of calorie load and consumption of different foods types had profound influences on diurnal cycles of gut bacteria. This correlates with the propensity towards fat production in the host. In September last year I decided to try aspects of this new knowledge out on myself. I had reached 205 lbs (93 kg), which at 6 foot (1.83 m) tall meant that I was only considered ‘overweight’, but that was enough for me. As well as changing other aspects of my lifestyle, including near complete abstinence in alcohol and soda, I started to consume more calories during breakfast, including more animal protein and fat, and then a salad at lunch and a normal meal during the evening. I suddenly found myself with more energy and enthusiasm for activity, and so I started to walk about 5 miles (8 km) a day. Over the following 12 weeks I lost 40 lbs (18 kg).

I had affected real change in my physiology, wardrobe (none of my suits fitted me anymore!) and subsequently my lifestyle. I did this by testing a series of hypotheses that can be summed up as ‘If I alter factors x, y and z, then I will lose weight’. To measure my experiment, I started to create a metric for the relevant variables in my life, such as calorie intake, calorie burn, activity, etc. Monitoring my activity was aided by taking the advice of Ewan Birney (@ewanbirney), and buying a FitBit, which measures steps and provides an app and website to help track the foods and calories you eat, water you drink, calories you burn, etc. A similar, albeit more comprehensive app was recently demonstrated in David et al’s [3] work on tracking the fecal microbiome, where the participants monitored some 300 or more variables daily! By monitoring the progress of my little experiment, I found it much easier to keep track of the results, which helped me to maintain momentum.

Research also resulted in my family and I rescuing a dog. Dogs can have a profound influence on microbial sharing by the occupants in a house [4], as well as reducing asthmatic attacks in mouse models [5]. When we performed our own study, employing longitudinal sampling of homes, it became apparent that dogs supercharged the network of transmission routes that enable bacteria to move around a house [6]. When I discussed these results with my wife, over 18 months prior to publishing them, we made an immediate decision adopt a dog; Captain Beau Diggly is now a firm part of our lives, and helps me to keep fit as well as maintaining an outdoor lifestyle.

I am following a long tradition (for example, Barry Marshall’s somewhat apocryphal experiments with *Helicobacter*), whereby scientists resort to performing experiments on themselves. By buying a dog, my wife and I have also started an experiment, albeit a rather innocuous one, on our family. While many of the changes we have wrought in our lives are probably common sense, we find it easier to accept them when we have evidence to back up our assumptions about their efficacy.

Science is not belief; anyone who thinks it is doesn’t really understand it. That doesn’t mean that people don’t take things on faith from time to time. I was at a conference in Egypt in April 2012, and I attended a ‘Nobel Prize Speaker’ session. The first speaker expounded the common spoken belief that young people should never take things on faith, that you should question everything, and I found myself agreeing with this rhetoric. However, then the second speaker fundamentally disagreed, and asked whether the first speaker would not trust the pilot of the plane he flew to the meeting on, to do their job? Whether or not the other speaker had a pilot’s license, I am sure he never asked to see that of the pilot flying that aircraft. He accepted on faith that the pilot knew what he was doing, and could fly the plane.

When not an expert in a particular area of knowledge, we often accept that a report we read in a newspaper is appropriately representative of the state of knowledge about that area. I don’t pretend to know enough about high-energy particle physics, and therefore am quite willing to accept that an expert in high-energy particle physics might know what they are talking about, at least far more than I do. While we continually apply the scientific method to problem solve the world around us, we are also bound to trust some of the myriad information we consume on a day-to-day basis, without rigorously questioning it.

As scientists we must remember that this trust that we place in others, is also the same trust that people place in us. Therefore, scientists and journalists alike have an important responsibility, to make sure that the communication of scientific work and results is appropriate and does not lead to ‘citizen scientists’ performing inappropriate experiments on themselves. When I performed my little experiments, I did so from a position of knowledge and expertise; also the approaches I chose to explore had an extremely low probability of resulting in bad consequences for either my family or myself. While I have often reviewed work on animal studies that demonstrate the efficacy of fecal microbiome transplant or probiotics to influence specific gastrointestinal or neurological conditions, I have not, and will not be, testing these on either my family or myself, unless the alternative had a significantly higher probability of a bad outcome.

Science changes and shapes our lives in every way, from the innate decisions we make every day, to purposeful striving for new discoveries. It is a powerful tool that can be wielded for benefit or maleficence, and constantly try to strive for the former.
